# A phase III randomized trial of weight loss to reduce cancer-related fatigue among overweight and obese breast cancer patients: MEDEA Study design

**DOI:** 10.1186/s13063-022-06090-6

**Published:** 2022-03-04

**Authors:** Antonio Di Meglio, Elise Martin, Tracy E. Crane, Cecile Charles, Aude Barbier, Bruno Raynard, Anthony Mangin, Olivier Tredan, Carole Bouleuc, Paul H. Cottu, Laurence Vanlemmens, Carine Segura-Djezzar, Anne Lesur, Barbara Pistilli, Florence Joly, Thomas Ginsbourger, Bernadette Coquet, Iris Pauporte, Guillemette Jacob, Aude Sirven, Julia Bonastre, Jennifer A. Ligibel, Stefan Michiels, Ines Vaz-Luis

**Affiliations:** 1 INSERM Unit 981–Molecular Predictors and New Targets in Oncology, Gustave Roussy, Villejuif, France; 2 Gustave Roussy, Villejuif, France; 3 University of Arizona, Tucson, AZ USA; 4 Centre Léon Berard, Lyon, France; 5 Institut Curie, Paris, France; 6 Centre Oscar Lambret, Lille, France; 7 Centre François Baclesse, Caen, France; 8 Institut de cancérologie de Lorraine, Nancy, France; 9 CAMI Sport & Cancer, Paris, France; 10 Ligue contre le cancer-France, Paris, France; 11 Seintinelles, Paris, France; 12 UNICANCER, Paris, France; 13 Service de Biostatistique et d’Epidémiologie, Gustave Roussy, Oncostat U1018, Inserm, University Paris-Saclay, Equipe labellisee Ligue Contre le Cancer, Villejuif, France; 14 Dana-Farber Cancer Institute, Boston, MA USA

**Keywords:** Breast cancer, Cancer-related fatigue, Body mass index, Overweight, Obesity, Weight loss, Survivorship

## Abstract

**Background:**

Elevated body mass index (BMI) represents a risk factor for cancer-related fatigue (CRF). Weight loss interventions are feasible and safe in cancer survivors, leading to improved cardio-metabolic and quality of life (QOL) outcomes and modulating inflammatory biomarkers. Randomized data are lacking showing that a lifestyle intervention aimed at weight loss, combining improved diet, exercise, and motivational counseling, reduces CRF. Motivating to Exercise and Diet, and Educating to healthy behaviors After breast cancer (MEDEA) is a multi-center, randomized controlled trial evaluating the impact of weight loss on CRF in overweight or obese survivors of breast cancer. Herein, we described the MEDEA methodology.

**Methods:**

Patients (*N* = 220) with stage I–III breast cancer and BMI ≥ 25 kg/m^2^, within 12 months of primary treatment, and able to walk ≥ 400 m are eligible to enroll. Participants are randomized 1:1 to health education alone vs. a personalized telephone-based weight loss intervention plus health education. Both arms receive a health education program focusing on healthy living. Patients in the intervention arm are paired with an individual lifestyle coach, who delivers the intervention through 24 semi-structured telephone calls over 1 year. Intervention goals include weight loss ≥ 10% of baseline, caloric restriction of 500–1000 Kcal/day, and increased physical activity (PA) to 150 (initial phase) and 225–300 min/week (maintenance phase). The intervention is based on the social cognitive theory and is adapted from the Breast Cancer Weight Loss trial (BWEL, A011401). The primary endpoint is the difference in self-reported CRF (EORTC QLQ-C30) between arms. Secondary endpoints include the following: QOL (EORTC QLQ-C30, -BR45, -FA12), anxiety, and depression (HADS); weight and BMI, dietary habits and quality, PA, and sleep; health care costs (hospital-admissions, all-drug consumption, sick leaves) and cost-effectiveness (cost per quality-adjusted life-year); and patient motivation and satisfaction. The primary analysis of MEDEA will compare self-reported CRF at 12 months post-randomization between arms, with 80.0% power (two-sided *α* = 0.05) to detect a standardized effect size of 0.40.

**Discussion:**

MEDEA will test the impact of a weight loss intervention on CRF among overweight or obese BC survivors, potentially providing additional management strategies and contributing to establish weight loss support as a new standard of clinical care.

**Trial registration:**

ClinicalTrials.gov NCT04304924

## Background

Over 80% of patients with early-stage breast cancer can nowadays expect long-term disease-free intervals after primary treatment. As a result, breast cancer survivors currently make up a substantial proportion of the population, as almost three million women in the United States of America (USA), two million in Europe, and 250,000 in France live with a history of breast cancer [1–3]. Improved survival rates have led to the emergence of a number of new, often unmet, clinical needs in survivorship care. Particularly, those related to the management of late and chronic side effects of cancer therapies and treatment-related symptoms represent key priorities [4].

Cancer-related fatigue (CRF) is one of the most common and persistent *sequelae* of breast cancer treatments [5], with a prevalence reaching 30–40% 1 year and up to 20% 10 years after treatment completion [6–9]. CRF is a complex and multifaceted syndrome, described as heavily distressing and impactful from a patient’s perspective. Its subjective expressions not only include generalized weakness and physical manifestations, but also emotional lability and loss of capacity to concentrate, which can interfere with normal functioning in a multitude of life domains spanning from usual daily activities, job tasks, and social relationships [8, 10–17].

### Excess body weight and CRF

The epidemic of overweight and obesity is a relevant public health issue in the general population worldwide, increasingly among breast cancer patients. Data suggest that up to 75% of women in the USA and 50% in Europe are overweight or obese at the time of breast cancer diagnosis [18–20], and additional weight gain after often results from breast cancer treatments [21–23].

Evidence has demonstrated a link between obesity and CRF, with data showing that elevated body mass index (BMI) represents a risk factor for CRF [11, 13, 24–29]. In a longitudinal study of women with early-stage breast cancer, BMI was significantly associated with CRF at the 42-month post-treatment assessment [11], and higher BMI also emerged as an independent predictor of membership to the high-level fatigue group in a study evaluating patterns of CRF after breast cancer treatment [24]. BMI significantly predicted chronic and persistent CRF for up to 2.5–7 years post-breast cancer diagnosis [25]. In addition, among white women with breast cancer enrolled in the Women’s Healthy Eating and Living (WHEL) Study, obesity was associated with significantly worse vitality (i.e., fatigue), compared to non-obese status [26, 27]. More recently, higher BMI was a risk factor for severe CRF in the prospective multicenter CANTO cohort study [6]. 

### Other risk factors and correlates of CRF

Previous literature highlighted several other correlates of CRF, including biobehavioral, psychological, and biological factors [13]. Relevant risk factors for the development and persistence of CRF among cancer survivors include pre-treatment fatigue, emotional distress (anxiety and depression), sleep problems, and physical inactivity and sedentary behavior [9, 13, 30, 31]. Concomitant medical conditions, comorbidities, inadequate nutrition, and symptoms such as uncontrolled pain and persistent sleep problems can also contribute to CRF [6, 30, 31].

Furthermore, although the etiology and biological substrate of CRF is not completely understood, a positive association between cytokine deregulation and increased CRF has been demonstrated. Survivors with persistent CRF have indeed been found to have increased expression of genes encoding pro-inflammatory markers or other mediators of immunologic activation [13, 32–36]. Chronic inflammatory alterations are common in individuals with higher adiposity. In obese individuals, the adipose tissue is expanded and reprogrammed, with specific metabolic activations leading to inflammatory changes in the local *milieau*. Additional interactions occur between local microenvironment and dysregulation in systemic biology, promoting a generalized inflammatory activity [37]. In this context, the enhanced pro-inflammatory cytokine network existing among individuals with higher BMI may be in part responsible for different alterations linked to CRF, including cellular immune system and neuroendocrine dysregulation [13].

### Weight loss interventions among breast cancer survivors

A number of studies looked at the feasibility and benefits of weight loss interventions in cancer survivors [38–44]. In-person or remotely delivered weight loss interventions are now deemed feasible and also safe in breast cancer survivors with excess body weight.

Examples of completed studies of large lifestyle interventions conducted in the USA among overweight or obese breast cancer survivors include the Lifestyle Intervention Study for Adjuvant Treatment of Early breast cancer (LISA) and the Exercise and Nutrition Enhance Recovery and Good Health for You (ENERGY) studies [45, 46]. These individualized interventions incorporating goals of caloric deficit and increased PA led to significant and greater weight loss across BMI strata and showed great potential in improving a number of outcomes for breast cancer survivors compared to delivery of general health and weight loss information alone. For example, a mean weight loss of 4–5 kg in the LISA intervention and of 6% of baseline in the interventional arm of ENERGY were linked to a more likely preservation of physical function, greater increases in physical condition, and improvement in a multitude of functional and symptom domains [45, 46].

More recently, the Alliance for Clinical Trials in Oncology Breast Cancer Weight Loss Trial, also known as A011401 (BWEL, NCT02750826, PI Jennifer A. Ligibel) started in the USA and Canada. BWEL is a National Cancer Institute-funded phase III randomized trial evaluating the effect of a weight loss program on cancer recurrence among over 3000 overweight and obese women with stage II to III breast cancer across over 1000 institutions. The BWEL weight loss intervention is standardized using the approaches developed as part of the Diabetes Prevention Program (DPP) [45] and the LISA study, focusing on diet, PA, and motivational factors [47]. BWEL is primarily testing the impact of weight loss on invasive disease-free survival (IDFS) among overweight (BMI ≥ 27 kg/m^2^) patients with stage II–III hormone receptor (HR)-positive, human epidermal growth factor receptor (HER)2-negative breast cancer. A subset study within BWEL will also assess the impact of the intervention on health behaviors and PROs, including CRF [48].

These interventional weight loss studies also provide data demonstrating the feasibility of conducting a lifestyle intervention for cancer survivors in a cooperative group setting.

### Study rationale

Weight loss has been shown to have several health benefits for cancer survivors with elevated BMI, such as leading to a better quality of life and lower rates of cardio-metabolic comorbidities including heart disease and diabetes [49]. Studies also suggest that there is rationale to test intentional weight loss during the early survivorship period as a strategy to mitigate CRF, often developing as one of the many downstream *sequelae* of primary breast cancer treatment. Recently, observational evidence from the French CANcer TOxicity (CANTO) Study showed that compared to the pre-treatment period and respective to women whose weight increased or remained stable, obese women who lost weight reported either improvements or more favorable variations in PROs for several domains of quality of life, including CRF [50, 51]. Much remains to be learned about the relationship between weight loss and CRF, including about the biologic changes that could mediate the relationship between obesity, weight change, and CRF. Previous studies suggested that weight loss may determine a modulation in inflammatory mediators, including leading to changes in levels of IL-6, TNF-α, and C-reactive protein, which had been associated with increased CRF [13, 52], and therefore contribute to CRF mitigation.

Motivating to Exercise and Diet, and Educating to healthy behaviors After breast cancer (MEDEA) is a randomized controlled trial (RCT) building on the personalized weight loss intervention of the BWEL study. Specifically, MEDEA (1) focuses on overweight and obese adult patients with a BMI ≥ 25 kg/m^2^ recruited after their initial breast cancer (stages I–III, HR±/HER2±) treatment; (2) implements an adapted version of the BWEL intervention that includes modifications to the length and content of the intervention, as well as language and substantial cultural adaptation; (3) primarily aims to evaluate the impact of the personalized telephone-based weight loss intervention on post-treatment CRF (and secondarily on quality of life and several other general and breast cancer-specific outcomes); and (4) may help develop a model for trials testing the impact of lifestyle changes on outcomes of breast cancer survivors outside the USA.

The MEDEA Study builds on the hypothesis that a combined lifestyle intervention of weight loss, incorporating multiple components of PA, nutritional counseling, and motivational support improves CRF among overweight and obese survivors of breast cancer. The mechanisms through which such intervention may reduce CRF are several, including that interventional components are able to target or modulate multiple contributing factors for CRF. Among the components of the MEDEA intervention, PA is the most studied and effective strategy for reducing CRF both during and after primary breast cancer treatment, as demonstrated by a number of clinical trials and metanalyses. Evidence also suggests that PA may help reduce physical dysfunction, emotional distress, pain, and sleep problems, which are frequent correlates of CRF, and act on the modulation of inflammatory mediators of CRF. In addition, nutritional counseling, while targeting caloric restriction and setting daily caloric intake limits, aims at maintaining a high-quality diet that can help identify and correct nutritional imbalances that can be associated with elevated CRF. Finally, motivational support may activate patient empowerment, allow to focus on intervention goals, and help reduce maladaptive thoughts, which are common in patients with CRF [30, 31, 53–56].

In this manuscript, we present the MEDEA Study methodology.

## Methods

### Study objectives

#### Primary objective

The primary objective (objective 1) of the MEDEA trial is to compare the effect of a personalized telephone-based weight loss program based on motivational coaching, exercise, and diet versus a standard health educational program control on CRF of overweight or obese breast cancer patients (reported by patients using the European Organisation for Research and Treatment of Cancer (EORTC) Quality of life Questionnaire (QLQ)-C30) [57, 58].

#### Secondary objectives

Secondary objectives of MEDEA include the following: secondary objective 1—the evaluation of the impact of the weight loss intervention on (1.1) specific CRF dimensions, including physical, emotional, and cognitive CRF; (1.2) QOL, including domains such as global health status, emotional, social, role, cognitive function, insomnia, pain, body image, and systemic therapy side effects; (1.3) sleep quality; and (1.4) anxiety and depression, weight and BMI, dietary habits and quality, and PA; secondary objective 2—the dissemination/implementation of the intervention, adapted from a US setting, in the French health care setting; secondary objective 3—health care costs (hospital admissions, all-drug consumption, sick leaves), cost-effectiveness (cost per quality-adjusted life-year QALY), and patient motivation and satisfaction; and secondary objective 4—adverse musculoskeletal events including fractures, sprains, tendon, or ligament injuries, and orthopedic surgeries are assessed (safety).

#### Exploratory objectives

MEDEA also has the following exploratory objectives: exploratory objective 1—assess the impact of the weight loss intervention on cancer-related outcomes (IDFS and overall survival [OS]); exploratory objective 2—assess the adherence to intervention by qualitative and quantitative evaluation. For the qualitative assessment, focus groups will be performed with selected study participants to understand barriers and facilitators to uptake and adherence to the intervention, including satisfaction and suggestions for improvement regarding the weight loss program. Focus groups are envisioned at least once halfway through the intervention (month [M] 6) and once at the completion of the intervention (M12). Table 1 summarizes MEDEA Study outcomes.

**Table 1:** MEDEA Study outcomes, measures, and assessment

Outcome type	Outcome		Measure	Type of assessment	Schedule of data collection			
					Baseline, M0	M6	M12	M18
**Primary**	**Cancer-related Fatigue**	**Global fatigue**	EORTC QLQ-C30^1^	Self-reported	X	X	X	X
**Secondary**	**Cancer-related Fatigue**	**Fatigue dimensions: physical, emotional, cognitive**	EORTC QLQ-FA12^2^	Self-reported	X	X	X	X
	**Quality of life, anxiety, and depression**	Other quality of life domains*	EORTC QLQ-C30^1^	Self-reported	X	X	X	X
			EORTC QLQ-B45^3^	Self-reported	X	X	X	X
		**Anxiety/Depression**	HADS^4^	Self-reported	X	X	X	X
	**Health behaviors**	**Dietary habits**	Food questionnaire^5^	Self-reported	X	X	X	X
		**Physical activity**	WHO GPAQ-16^6^	Self-reported	X	X	X	X
		**Intensity of physical activity**	Actigraph Accelerometer^7^	Device-based	X	X	X	X
		**Energy expenditure**	Actigraph Accelerometer^7^	Device-based	X	X	X	X
		**Daily steps**	Actigraph Accelerometer^7^	Device-based	X	X	X	X
	**Weight and body composition**	**Weight**	Clinical visit	Hospital-based	X	X	X	X
		**Body mass index**	Clinical visit	Hospital-based	X	X	X	X
		**Waist and hip circumference**	Clinical visit	Hospital-based	X	X	X	X
	**Cost-effectiveness**	**Length of all-cause hospitalizations**	Direct medical costs	**	Throughout the whole study duration			
		**Drug consumption:** antidepressants, anxiolytics, pain-killers, and anti-inflammatory drugs (name, start date, duration of use)	Direct medical costs	**	Throughout the whole study duration			
		**Sick leave** (number, duration, and reason)	Indirect costs	**	Throughout the whole study duration			
		**Utility/QALY**	EuroQol 5D 3L^8^	Self-reported	X	X	X	X
**Exploratory**	**Survival outcomes**	**Invasive DFS**	Relapses	**	Throughout the whole study duration			
		**OS**	Death	**	Throughout the whole study duration			
	**Satisfaction and adherence**	Motivation and satisfaction with the intervention	Qualitative	Focus group	Halfway through the intervention (month 6) + once at intervention completion (month 12)			
		% delivered/planned phone calls % patients who achieve intervention goals % phone calls/total by patient/coaches	Quantitative	Centrally assessed (study manager)	End of study			

^1^Aaronson NK, Ahmedzai S, Bergman B, et al. The European Organization for Research and Treatment of Cancer QLQ-C30: a quality-of-life instrument for use in international clinical trials in oncology. J. Natl. Cancer Inst. 1993;85(5):365–376

^2^Weis J, Tomaszewski KA, Hammerlid E, et al. International Psychometric Validation of an EORTC Quality of Life Module Measuring Cancer Related Fatigue (EORTC QLQ-FA12). J. Natl. Cancer Inst. 2017;109(5)

^3^Bjelic-Radisic V, Cardoso F, Cameron D, et al. An international update of the EORTC questionnaire for assessing quality of life in breast cancer patients: EORTC QLQ-BR45. Ann. Oncol. 2020;31(2):283–288

^4^Zigmond AS, Snaith RP. The hospital anxiety and depression scale. Acta Psychiatr. Scand. 1983;67(6):361–70

^5^Gazan R, Vieux F, Darmon N, Maillot M. Structural Validation of a French Food Frequency Questionnaire of 94 Items. Front. Nutr. 2017;4:62

^6^Global Physical Activity Questionnaire Analysis Guide GPAQ Analysis Guide Global Physical Activity Questionnaire (GPAQ) Analysis Guide

^7^Esliger DW, Tremblay MS. Technical reliability assessment of three accelerometer models in a mechanical setup. Med. Sci. Sports Exerc. 2006;38(12):2173–2181

^8^EQ-5D

*Including: Global health status, physical, emotional, social, role, cognitive function, Pain, Insomnia, Nausea/Vomit, Dyspnea, Appetite Loss, Constipation, Diarrhea, Financial difficulties, Body Image, Future perspective, Sexual function and enjoyment, systemic therapy side effects, arm and breast symptoms, endocrine therapy, skin, and sexual symptoms

**National health, insurance, hospital-based records, ad hoc questionnaire. *M* month

### Study design

**Fig. 1:**
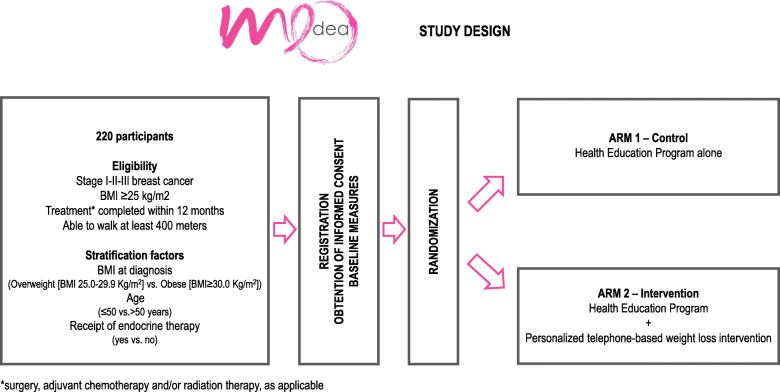
MEDEA Study design. BMI, body mass index

MEDEA is a RCT evaluating the effect of health education alone vs. weight loss intervention plus health education on CRF in overweight and obese women with early-stage breast cancer. Patients are offered to participate in this study during routine consultations. Participants are randomized to a standard health education program (arm 1 control) vs. a 1-year personalized telephone-based health education weight loss follow-up (arm 2 interventional). A permuted block randomization is applied in a 1:1 randomization ratio using a block size of 4. Stratification occurs on the basis of BMI at diagnosis (overweight [BMI 25.0–29.9 kg/m^2^] vs. obese [BMI ≥ 30.0 kg/m^2^]), age (≤ 50 vs. > 50 years), and receipt of endocrine therapy (yes vs. no) (Fig. 1).

To ensure allocation concealment, patients who have signed the informed consent and fulfilled all eligibility criteria will be randomized directly online using the Trialmaster randomization module. Patients are randomized for either arm after the accrual visit. Patients are enrolled through local clinic sites, and the weight loss intervention is delivered remotely by trained study coaches. Target enrollment is 220 patients.

### Eligibility

#### Inclusion criteria

Patients are eligible if they have histologically confirmed stage I–III breast cancer without evidence of distant metastatic or locally recurrent disease at inclusion. Participants with a history of invasive breast cancer in the 5 years prior to study registration other than the current diagnosis are ineligible for enrollment. Prior ductal carcinoma in situ (DCIS) at any time does not make a patient ineligible. Subjects must have a BMI ≥ 25 kg/m^2^, either objectively assessed at the time of eligibility evaluation or reported in the medical records within 56 days prior to study registration. All adjuvant or neoadjuvant chemotherapy, radiation, and surgery must have been completed no more than 12 months before enrollment. Patients may have breast reconstruction during protocol participation, but definitive breast cancer surgery must be completed prior to registration. Biologic therapy, hormonal therapy, and bisphosphonates are acceptable to be ongoing while on study. Participants should have an ECOG Performance Status of ≤ 1 and self-reported ability to walk at least 400 meters (at any pace). Each participant should understand, sign, and date the written informed consent form prior to any protocol-specific procedures and be able and willing to comply with the study visits and procedures as per protocol. Patients must be able to read and speak French to be eligible for inclusion.

#### Non-inclusion criteria

Participants in both arms are allowed to pursue weight loss and PA programs on their own, but participation in another weight loss, PA, or dietary intervention clinical trial at a time is not allowed. Comorbid conditions, such as other malignancy, diabetes, inflammatory bowel disease, history of severe cardiovascular, respiratory, or musculoskeletal disease that would preclude adherence to the study diet or PA program or significantly affect the physical status or the ability to give an informed consent make a patient ineligible for enrollment. Secondary overweight or obesity must not be documented or suspected at the time of enrollment. Chronic consumption of corticosteroids or self-reported pregnancy or intent to become pregnant in the year after enrollment also represents ineligibility criteria.

#### Withdrawal criteria

Premature discontinuation of study intervention does not necessarily mean the patient prematurely discontinues its participation in the study. (1) Withdrawal criteria from study intervention. A patient will not receive any further study intervention if any of the following occurs: request from the patient not to receive further intervention; withdrawal of consent, or lost to follow-up; adverse events or any condition incompatible with the continuation of the study intervention according to the judgment of the investigator; any medical event requiring administration of an unauthorized concomitant treatment; pregnancy or intent to become pregnant; subject non-compliance to study procedures that in the investigator and/or sponsor judgment warrants withdrawal; study terminated by the sponsor; patients with disease recurrence at any site, or new invasive cancer diagnosis, can continue the intervention if they wish, and if it is considered appropriate by physician discretion. Patients who are withdrawn from further receipt of study intervention will continue to have *follow-up visits.* (2) Withdrawal criteria from the trial. Reasons for withdrawal from the trial may include lost to follow-up, consent withdrawn, disease recurrence or new invasive cancer diagnosis, and death.

### Study intervention

The MEDEA intervention is based on the telephone-based weight loss program that was standardized as part of BWEL [47].

#### Health education program (arms 1 and 2)

All patients in both the health education control and the intervention group receive a standardized Health Education Program focusing on healthy living. This includes a welcome letter, paper brochures, and links to websites focused on cancer-related topics. Covered themes include healthy eating, exercising, nutrition, and cancer.

In addition, all study participants receive a quarterly newsletter with additional materials, including invitations to join webinars/conferences that focus on breast cancer and other health topics (e.g., treatment updates in breast cancer, management of specific side effects, general cancer screening). Finally, patients are provided with updates about study and enrollment progress.

#### Weight loss intervention (arm 2)

Patients randomized in the interventional arm of MEDEA receive behavioral support to facilitate lifestyle change based on caloric restriction and increased PA, delivered remotely by a weight loss coach. Personalized coaching is crucial for the MEDEA intervention; therefore, participants are paired with an individual coach who works with them through all phases of the weight loss program. Lifestyle coaches are required to have expertise in diet/nutrition and received initial training in behavioral change, PA, and/or weight loss in cancer patients.

The behavioral change program is based on the social cognitive theory, which hypothesizes that the interactions between environmental, personal, and behavioral elements determine behavioral change [59].

The intervention is implemented through 24 semi-structured and standardized telephone calls, delivered over 1 year, supplemented by a detailed participant workbook that is mailed to participants after randomization. Participants are encouraged to review the content of the call beforehand using the workbook in order to facilitate the telephone interactions with their coach. Calls are scheduled at the participant’s convenience. Each call lasts approximately 30 min, although the first four calls last longer (up to 45–60 min). Calls are scheduled as follows: intensive phase (weeks 1–12; 12 weekly calls), consolidation phase (weeks 13–24; six bi-weekly calls), and maintenance phase (week 25–end of intervention; one monthly call). Every effort is made to ensure that a core of 16 calls (the minimum number required to ensure the success of the intervention) take place during the first 6 months and that the frequency is greatest early in the intervention, when participants are making the most rapid changes. However, the intervention is considered to be completed if at least 16 calls are completed over 1 year. Table 2 outlines the 24 telephone calls with respective thematic content. A call is considered completed if the specific thematic content is approached and delivered. Notwithstanding standardization of the intervention, because of the focus on behavioral change and not to increase patient burden, the study allows flexibility in the approach taken with individual participants. This flexibility is reflected in adjusting calls scheduling by accommodating, for example, for vacations or personal/family plans, and by tailoring thematic content. In general, if one or more calls are skipped, thematic contents regarding caloric intake and ways to improve PA are prioritized when calls are resumed, in order to reinforce key messages and facilitate participants to meet dietary and activity goals and make the changes recommended as part of the intervention.

**Table 2:** MEDEA telephone-based intervention sessions: schedule and thematic content

Intervention phase	Phone call	Week	Title	Thematic content
**Intensive phase:** weekly calls	**1**	1	Welcome to the MEDEA program	Introduction to program
	**2**	2	Getting started: tipping the calorie balance	Caloric restriction
	**3**	3	Not all fats are created equal: eat less of most, more of some	Caloric restriction
	**4**	4	Cutting calories by controlling your portions	Caloric restriction
	**5**	5	Move those muscles!	Physical activity
	**6**	6	Working with what’s around you: cue control	Behavioral support
	**7**	7	Problem solving	Behavioral support
	**8**	8	Being active: a way of life	Physical activity
	**9**	9	Healthy eating	Caloric restriction
	**10**	10	Healthy eating and breast cancer	Breast cancer and nutrition
	**11**	11	Preparing a better breakfast, lunch, and dinner	Caloric restriction and Behavioral support
	**12**	12	Summary and progress review	Summary and evaluation
**Consolidation phase:** bi-weekly calls	**13**	14	Talk back to negative thoughts	Behavioral support
	**14**	16	The slippery slope of lifestyle change	Behavioral support
	**15**	18	Stepping up your physical activity	Physical activity
	**16**	20	Ten ways to control your hunger	Behavioral support
	**17**	22	Handling holidays, vacations, and special events	Behavioral support
	**18**	24	Taking stock and celebrating your success	Summary and evaluation
**Maintenance phase:** monthly calls	**19**	28	Ways to stay motivated	Behavioral support
	**20**	32	Recovering from overeating	Behavioral support
	**21**	36	Weight loss review	Caloric restriction
	**22**	40	Adapting to long-lasting success	Behavioral support
	**23**	44	Preparing for what comes next	Summary and evaluation
	**24**	48	Congratulations! You finished the MEDEA program!	Summary and evaluation

Lifestyle coaches individualize the call content as necessary to address individual problems (ensuring that key aspects of each call are covered) and are trained to address motivational and emotional issues as they arise, partnering with participants to overcome barriers to success. Data on compliance, changes in diet, PA, and weight reported by participants are captured and discussed with coaches during the calls. Coaches prepare a brief written summary of motivational, behavioral, diet, and exercise issues discussed during each call.

A toolbox approach that allows for tailoring the intervention to the individual participant is available to facilitate reaching intervention goals and losing weight. Toolbox solutions also aim at optimizing the intervention to meet the needs of specific ethnic, socioeconomic, or other patient populations. Toolbox materials were partly developed by coaches during the preparation phase of the study and reviewed by the MEDEA Study expert board. Examples of MEDEA Toolbox solutions are listed in Table 3.

**Table 3:** Examples of content for MEDEA Toolbox approaches

**Eating patterns and meal plans**	- High-protein/low-carb eating pattern - Mediterranean eating pattern
**Cooking and shopping tips**	- Using your hands to estimate portions - Weighing and measuring foods - Food shopping on a budget - Commercial protein bars - Meal prep and batch cooking
**Activity and symptoms**	- Three examples of resistance training - 50 ideas to increase daily steps - Apps, videos, books, and tutorials to exercise - Being active with neuropathy - Being active with joint pain - Being active with lymphedema
**Miscellaneous**	- Sports and cancer - Managing weight when you quit smoking - “Use” your hunger

**Fig. 2:**
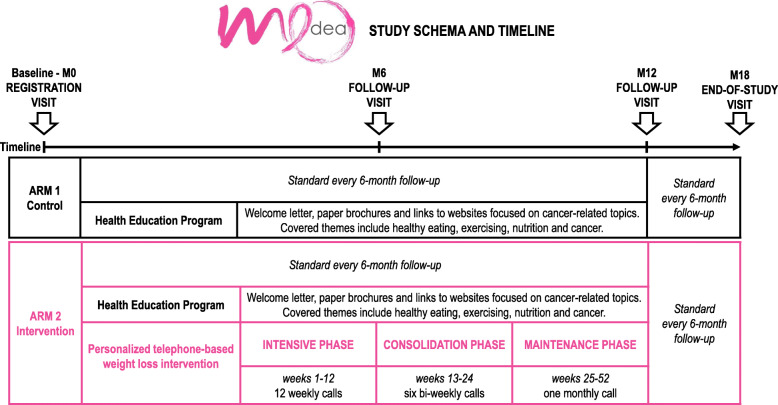
MEDEA Study schema and timeline. M, month

The MEDEA Study schema and timeline are displayed in Fig. 2.

#### Intervention goals

MEDEA Study goals include the following:

- Weight loss: weight loss ≥ 10% of baseline (0.5–1.0 kg per week) until a BMI no less than 21 kg/m^2^.
- Caloric restriction: caloric deficit of 500–1000 Kcal per day based on current weight and weight loss target. Recommended calorie levels are based on body weight and standardized tables: calorie levels range from 1200 to 1499 kcal/day for participants weighing < 110 kg at baseline and 1500–1800 kcal/day for individuals weighing ≥ 110 kg at baseline. Caloric goals are modified as necessary to assist participants in reaching their goals. The basic diet used in the MEDEA materials supports a low-fat, high-fruit and vegetable diet.
- PA: the primary focus of the PA portion of the weight loss intervention will be on moderate-intensity aerobic activity. Gradual increase in moderate-intensity aerobic activity has a target goal of 150 min/week during the initial phase of the intervention and 225–300 min/week in the maintenance phase of the intervention.

#### Standardization of the intervention and quality control

Approaches to standardization of the intervention include the following: (1) A standardized training protocol for the coaches that was piloted in the BWEL study was developed by the MEDEA and BWEL investigators. Training involved the use of printed materials (e.g., protocol, coach’s manual, and a sample of call scripts) and in-person activities (e.g., behavioral/motivational interviewing techniques, role playing, review of telephone tapes). (2) Standardized supervision of coaches: regular meetings with coaches and the MEDEA Study board and coordinators are held on a weekly basis to review progress and troubleshoot problems associated with the delivery of the intervention. Coaches have the ability to discuss their calls summary and to receive feedback on relevant aspects of their interactions with study participants. Regular participation of lead nutrition, exercise, and behavioral experts to MEDEA meetings is assured to address specific issues. (3) Recording and centralized review of 5% randomly selected phone calls: these calls are reviewed by the MEDEA Study board and coordinators with the coaches for adherence to the study intervention, and standardized feedback is provided to the individual coaches. (4) Centralized calculation of individual weight. (5) Centralized response to diet and PA problems arising between telephone calls. Participants are provided with contacts of the MEDEA Study coordinators, to alert study personnel of issues that develop during participation in the intervention. Medical problems are referred to the participants’ treating oncologist or generalist. (6) Centralized feedback system: coaches have the ability to refer to the MEDEA team at any time for questions and to troubleshoot problems arising during the delivery of the intervention, through a centralized e-mail managed by a dedicated study manager.

### Study measures

The MEDEA Study assessments include a baseline (M0) assessment (at time of enrollment), two “on-health education-intervention visits” (M6 and M12), and a final follow-up visit (M18). Participants undergo study visits at their local site. Collected measures include CRF, quality of life, depression and anxiety, health behaviors, anthropometric measures, cost-effectiveness measures, assessment of disease status and adverse events, adherence, and satisfaction with the study intervention. Details about the study measure type and frequency of assessment are reported in Table 1.

All patients in MEDEA are provided with Actigraph accelerometers [60], recording the duration and intensity of PA. Readings are obtained centrally through the use of the ACTILIFE software [61]. Participants do not receive any feedback from the accelerometer unit, regardless of the study arm. Participants wear the accelerometer for 7 days at all four time points (M0, M6, M12, and M18). Accelerometers are mailed to participants by the study manager along with an instruction sheet regarding its use and a diary form to indicate the dates that the accelerometer was worn. Participants return the accelerometer to Gustave Roussy site along with a pre-addressed return mailer. For the first visit, they wear the accelerometer before the start of the intervention if randomized to arm 2. For subsequent assessments, a pre-paid mailer is included in the packet so that participants can directly return materials to Gustave Roussy. Dietary assessment is performed using a 94-item food frequency questionnaire [62]. Weight assessments are performed by health care professionals in the clinic during follow-up visits.

Direct medical costs and indirect costs will be assessed from the perspective of the French national health insurance using data linkage to SNDS (“Système national des données de santé” – National Health data system [63]) database for each patient (indirect matching using the date of birth, zip code of the patient’s residence, type/date/hospital identification code for any breast cancer treatment, health insurance characteristics). Resource use includes all-cause hospitalizations (distinguishing between breast cancer-related hospitalizations and hospitalizations for other causes), all drugs consumption, and sick leaves. QALYs are measured using utility values derived from the Euroqol-5D (EQ-5D-3L) [64] at all four time points (M0, M6, M12, and M18).

Participants in both study arms are asked about the following musculoskeletal events during each follow-up visit: fractures, sprains, tendon or ligament injuries, and orthopedic surgeries. Non-solicited adverse events such as post-surgical wound infections or side effects from hormonal therapy such as hot flashes are not required to be reported for this trial.

Adherence to the intervention is measured by the number of completed phone calls/total number of calls scheduled. To define the intervention as completed, a minimum of 16 calls have to be completed. Satisfaction with the intervention will be also assessed with qualitative analyses and dedicated focus groups.

IDFS is defined as the time from randomization to any one of the following: distant, invasive, or locoregional recurrence; ipsilateral or contralateral invasive breast cancer; second primary invasive cancer (non-breast cancer other than basal or squamous cell carcinoma of the skin that has been adequately treated, and carcinoma in situ of the cervix); or death from any cause. Overall survival is defined as the time from randomization to death from any cause. Patients who withdraw consent to be followed are censored at that time point, and patients who are lost-to-follow-up are censored at the time point they were last determined to be disease-free.

### Implementation of study procedures

Patients who have completed primary treatment (including adjuvant or neoadjuvant chemotherapy, radiation, and surgery) within 12 months and satisfy all the study eligibility criteria are offered study participation by the treating clinician during a standard follow-up visit at a MEDEA participating institution. Signature to study consent is obtained, and patients enter the study. The patient then meets with the study nurse, who collects the required baseline study measures including baseline objective anthropometric measures. Randomization then occurs, and patients are assigned either to the control or to the intervention arm. Patients are then contacted by the study coordinator to go over study material and study intervention schedule if applicable. If a patient is randomized in the intervention arm, they are paired with an individual lifestyle coach, and the study coordinator schedules telephone calls according to the study schema, timeline, and availability of time slots for coaches. The study coordinator oversees the study procedures and assures optimal scheduling and troubleshoots organizational problems. Study materials are sent to patients both in the control and interventional arm, including materials of the Health Education Program and accelerometers for the initial M0 assessment. In addition, patients in the interventional arm receive the participant workbook as a support to the telephone-based sessions. Study procedures then follow the schedule outlined in Table 1 and Fig. 2. The trial is coordinated by Gustave Roussy. A project manager is responsible for all the administrative aspects of the trial, including coordination between centers and between patients and coaches (including scheduling of calls and mailing materials to patients).

### Ancillary and post-trial care

Patients eligible for the MEDEA Study diagnosed with stage I–III breast cancer have completed their primary treatment. After completion of study follow-up (i.e., M18), patients go back to standard-of-care follow-up in the setting of regular surveillance for early-stage breast cancer.

### Data collection, management, and auditing

The Biostatistics and Epidemiology Unit at Gustave Roussy implemented an electronic case report form (eCRF) to allow secure online-direct data collection, using the Trialmaster software suite. Each user has personal identifiers (user ID/password), and data access is strictly limited according to profiles: (1) hospital clinical research assistant (CRA), allowing data entry on the eCRF; (2) data manager, allowing the first data monitoring, perform consistency checks, and edit requests for clarification addressed to the investigator or hospital CRA; and (3) investigator profile, enabled to sign and validate the data electronically. An e-learning is mandatory to access the eCRF. The password is configured when the profile is activated and must be changed every 6 months. For each patient included, the eCRF has to be completed by hospital CRA and signed by a study investigator. An audit trail within the system tracks all the changes made to the data. The data collected through the eCRF are the data source for the analysis. Data collected will be managed in the Biostatistics and Epidemiology unit at Gustave Roussy. Standard institutional practices will be followed to maintain the confidentiality and security of data collected in this study. A copy of the consent form and documentation of consent will be stored in a locked cabinet or an encrypted, password-protected computer drive. All protected health information collected from the study eCRF will be encrypted and password-protected. Questionnaires filled out on paper are stored in a locked cabinet in a secured office in Gustave Roussy. Data will be stored until data analysis is complete and then the data is transferred to a centralized repository. Access to the repository will be limited to the principal investigator, co-investigators, and associates from the original study team. Future studies requesting the use of the data must either be related to the original research study or will require separate IRB approval. The data collected through the eCRF are the data source for the analysis. In order to guarantee the authenticity and the credibility of the data in conformity with good clinical practices, auditing and quality assurance systems include (1) study management in accordance with standardized procedures at Gustave Roussy, (2) quality control performed by the CRA, and (3) auditing of investigating centers. Particularly, it is the responsibility of the CRA (i) to check that the investigator’s file is correctly and regularly updated; (ii) to verify the signatures and validity of consent forms, fulfillment of eligibility criteria, validity of evaluation criteria, and adverse events; and to (iii) assure that reporting requirements are met. Regular meetings (at least monthly) with the investigator from the coordinating center and from other participating centers and study coordinator assure review of study procedures, periodic updates on study progress (including number of patients enrolled on-study as compared to expected numbers), and troubleshoot procedural problems.

### Statistical considerations

Previous meta-analyses indicated that exercise was more effective than control in reducing CRF, with a standardized effect size ~ 0.40 [31, 65–67], but there are limited data on the effect of lifestyle interventions aimed at weight loss on CRF among overweight or obese patients. To detect an effect size of 0.40, a sample size of 101 evaluable patients per arm will provide 80.0% power using a two-sided α = 0.05, two-sample test. The primary test statistic for inference is the time-by-treatment interaction effect in a linear mixed model. This effect size (0.40) is considered as small to moderate by Cohen [68]. Taking some potential drop-out into account, the final sample size for this study is inflated to 220 patients. The primary comparison of CRF among intervention assignments will be performed in the intent-to-treat population of all randomized patients (i.e., all analyses will retain each participant in the group he was randomly assigned regardless of missing data or drop-out status).

The primary analysis of MEDEA will compare the primary endpoint of CRF scores at the M12 post-randomization time point between arms. This comparison between arms will employ a linear mixed model with random patient effect using time as a categorical variable, treatment arm, and a treatment by time interaction with patient-reported CRF measured by appropriate EORTC QLQ-C30 subscale scores. In a sensitivity analysis, baseline characteristics will be added as fixed covariates to the model.

All other measures/time points/analyses will be considered secondary or exploratory analyses. Similar analyses as those specified for the primary endpoint will be performed for other scales. For binary endpoints, chi-squared tests will be used. Further analyses will include analyses of continuous endpoints using mixed-effects models and binary endpoints using generalized estimating equations. The analyses of objectives including IDFS as an endpoint are performed in an intention-to-treat manner.

For the primary analysis of MEDEA (primary endpoint of global CRF), two-sided *p*-values < 0.05 will be considered statistically significant. All other endpoints are considered secondary or exploratory, with no *p*-value adjustment for multiple testing. For interpreting the clinical significance of effects, 0.2, 0.5, and 0.8 standard deviation effects will be considered as small, moderate, and large, respectively [68].

### Trial status and data availability

The MEDEA Study received regulatory approval by the institutional review board of Gustave Roussy and by the National review board of France in February 2020. Recruitment was activated in June 2020. Accrual was completed in 2021, results will be available  in 2023.

Trial registration: ClinicalTrials.gov Identifier: NCT04304924. Registered on 12 March 2020, https://clinicaltrials.gov/ct2/show/NCT04304924 [69].

### Protocol amendments

The first study protocol was approved in January 2020. The protocol was then amended in July 2020 to accommodate the following changes: inclusion of EORTC QLQ-BR45 [70] and 94-item food frequency questionnaire [62] among study measures; removal of the initial 21-day time limit post-primary treatment for patient inclusion; possibility to include patients while on treatment with an investigational product that has fatigue as a known adverse effect

## Discussion

CRF is highly prevalent and persistent after breast cancer treatment, and increased BMI was associated in several studies with prevalent and chronic CRF among survivors of breast cancer. MEDEA will evaluate the impact of a personalized telephone-based weight loss intervention on post-treatment CRF, other quality of life domains, and several general and cancer-specific outcomes among overweight and obese breast cancer survivors in France.

Calorie restriction, while maintaining high diet quality, PA and behavioral counseling—promoting interactions between environmental, personal, and motivational elements determining behavioral change [59]—are the cornerstones of weight loss. Studies in non-cancer populations suggest that calorie restriction is essential in initial weight loss, while PA plays a greater role in weight maintenance. Studies also looked at the feasibility and benefits of weight loss interventions in cancer populations, comparing the efficacy of different strategies or assessing the combined impact of caloric restriction and PA on weight changes [38, 39]. Dietary change alone determined weight loss ranging from 2 to 10% of baseline body weight, while in interventions that included also a PA component weight loss ranged from 3.5–14% of baseline weight [38–45]. Weight loss of such magnitude seemed to be sustainable over time, and also led to significant improvements in PROs, particularly physical functioning, in breast cancer survivors [45].

Randomized trials were conducted or are currently ongoing evaluating the effects of lifestyle change after breast cancer diagnosis, including that of weight loss, increased PA, or dietary change, on outcomes such as recurrence or mortality [27, 47, 49, 71, 72]. Interventional trials evaluated the impact of PA and weight loss on a diverse array of outcomes for breast cancer survivors, also providing insight into the biologic mechanisms through which lifestyle factors may impact CRF and other quality of life domains among individuals with higher BMI. However, further research is needed to elucidate the impact of weight loss on frequently reported post-treatment symptoms such as CRF. MEDEA has several strengths and unique characteristics in the current context of studies testing weight loss interventions in cancer populations and has potential to expand the knowledge about the role of weight loss in managing CRF.

First, the MEDEA Study is based on a standardized and validated telephone-based weight loss program that is being used as part of the BWEL study [47]. Study materials were obtained and adapted from BWEL, including a standard health education program and a personalized telephone-based follow-up intervention, and underwent language adaptation from English to French to generate the MEDEA materials. A thorough review of BWEL materials was performed by a MEDEA Study expert board that includes medical oncologists, psychologists, nutrition, exercise, and behavioral counseling experts. The study board systematically included investigators from BWEL. A continuous exchange of intervention materials, tools, feedback, and expertise from the BWEL study ensured high standards in the process of conceptualization, funding acquisition, adaptation, and writing of the study protocol for MEDEA. This process lasted approximately 18 months and resulted in a simplified version of BWEL, including a 1-year intervention in MEDEA vs. a 2-year intervention offered in BWEL, and the thematic adaptation of intervention content to cultural, societal, and dietary habits of French participants. In addition, some of the tools used in BWEL were not implemented in MEDEA (i.e, distribution of scales and wearable activity sensors, meal replacement shakes, or measuring cups). Toolbox items were also adapted to reflect cultural preferences. Patient advocates were extensively involved during study preparation and review. Anti-cancer coalitions and patient groups including the “Ligue nationale contre le cancer [73]” and the “Seintinelles [74]” network actively participate in the dissemination of the study, including facilitating achieving adequate participant enrolment to reach the target sample size. Of note, MEDEA is a pragmatic trial with a shorter intervention and follow-up time compared to the BWEL study.

Second, the MEDEA trial develops in the setting of few randomized interventional trials testing weight loss interventions among cancer survivors in Europe. Examples of such trials include the German SUCCESS C trial and the Italian DIANA-5 study. While SUCCESS C will compare DFS in patients with a body mass index of 24–40 kg/m^2^ receiving a telephone-based individualized lifestyle intervention program aimed at moderate weight loss vs. general recommendations for a healthy lifestyle alone [71], DIANA-5 will assess breast cancer recurrence among participants randomly assigned to a lifestyle intervention focused on exercise and consumption of a Mediterranean, macrobiotic diet, or to a usual-care comparison group [72]. Both studies have a primary endpoint of cancer recurrence. Compared to these two studies, MEDEA specifically focuses on the impact of a personalized weight loss strategy on a primary endpoint of reducing post-treatment CRF.

Third, the results of MEDEA can have an impact at several levels. These include (a) on health care professionals, patients, and the general population, by (i) showing improvements of CRF and quality of life through a personalized weight loss program, (ii) providing better understanding of uptake of health behaviors and lifestyle interventions in France, and (iii) increasing awareness of personalized survivorship medicine; (b) on the medical research community, by (i) providing solid methodology about lifestyle interventions (ii) and opening the path for personalized survivorship research in other chronic diseases where weight loss could have a significant impact on patient outcomes; and (c) on French decision-makers and health authorities, by (i) providing scientific evidence and cost-effectiveness analyses results, (ii) implementing a personalized telephone-based weight loss program, (iii) producing clinical guidelines and policy recommendations for survivorship care, and (iv) identifying adequate care pathways models for the delivery of a personalized telephone-based health weight loss intervention. MEDEA has indeed the potential and ambition to prove that an inexpensive lifestyle strategy can be implemented in the French health care system, resulting in reduced utilization and costs of health care services. Finally, the adaptation of materials from the BWEL intervention to the MEDEA intervention was also purposely conceived to result in a readily disseminable intervention to broad clinical practice.

In conclusion, growing evidence supports the role of weight management, improved dietary quality, and PA in breast cancer prevention and control [75]. MEDEA will answer the important question of whether a personalized weight loss program is able to reduce CRF in post-treatment breast cancer survivors who are overweight or obese. If successful, MEDEA will help expand the currently available management strategies for an extremely common and distressful symptom such as CRF. More broadly, MEDEA will contribute to a global effort to establish weight loss support for overweight or obese breast cancer survivors as a new standard of clinical care.

## Data Availability

The data are not yet available for the MEDEA Study as the trial is still in progress.

## Abbreviations

BMI: Body mass index
BWEL: Breast Cancer Weight Loss trial
CANTO: CANcer Toxicity
CRF: Cancer-related fatigue
DCIS: Ductal carcinoma in situ
DPP: Diabetes Prevention Program
ECOG: Eastern Cooperative Oncology Group
ENERGY: Exercise and Nutrition Enhance Recovery and Good Health for You
EORTC: European Organisation for Research and Treatment of Cancer
IDFS: Invasive disease-free survival
LISA: Lifestyle Intervention Study for Adjuvant Treatment of Early breast cancer
M: Month
MEDEA: Motivating to Exercise and Diet, and Educating to healthy behaviors After breast cancer
OS: Overall survival
PA: Physical activity
PROs: Patient-reported outcomes
QALY: Quality-adjusted life-year
QLQ: Quality of life questionnaire
RCT: Randomized controlled trial
SNDS: Système national des données de santé
USA: United States of America
WHEL: Women’s Healthy Eating and Living Study
